# Unilateral versus bilateral robot-assisted rehabilitation on arm-trunk control and functions post stroke: a randomized controlled trial

**DOI:** 10.1186/1743-0003-10-35

**Published:** 2013-04-12

**Authors:** Ching-yi Wu, Chieh-ling Yang, Ming-de Chen, Keh-chung Lin, Li-ling Wu

**Affiliations:** 1 Department of Occupational Therapy and Graduate Institute of Behavioral Sciences, College of Medicine, Chang Gung University, Taoyuan, Taiwan; 2 Healthy Ageing Research Center, Chang Gung University, Taoyuan, Taiwan; 3 Department of Occupational Therapy, Kaohsiung Medical University, Kaohsiung, Taiwan; 4 School of Occupational Therapy, College of Medicine, National Taiwan University, Taipei, Taiwan; 5 Division of Occupational Therapy, Department of Physical Medicine and Rehabilitation, National Taiwan University Hospital, Taipei, Taiwan; 6 Department of Occupational Therapy, Mackay Memorial Hospital, Taipei, Taiwan

**Keywords:** Cerebrovascular accident, Rehabilitation, Robot, Kinematics, Motor control, Trunk compensation, Activities of daily living

## Abstract

**Background:**

Although the effects of robot-assisted arm training after stroke are promising, the relative effects of unilateral (URT) vs. bilateral (BRT) robot-assisted arm training remain uncertain. This study compared the effects of URT vs. BRT on upper extremity (UE) control, trunk compensation, and function in patients with chronic stroke.

**Method:**

This was a single-blinded, randomized controlled trial. The intervention was implemented at 4 hospitals. Fifty-three patients with stroke were randomly assigned to URT, BRT, or control treatment (CT). Each group received UE training for 90 to 105 min/day, 5 days/week, for 4 weeks. The kinematic variables for arm motor control and trunk compensation included normalized movement time, normalized movement units, and the arm-trunk contribution slope in unilateral and bilateral tasks. Motor function and daily function were measured by the Wolf Motor Function Test (WMFT), Motor Activity Log (MAL), and ABILHAND Questionnaire.

**Results:**

The BRT and CT groups elicited significantly larger slope values (i.e., less trunk compensation) at the start of bilateral reaching than the URT group. URT led to significantly better effects on WMFT-Time than BRT. Differences in arm control kinematics and performance on the MAL and ABILHAND among the 3 groups were not significant.

**Conclusions:**

BRT and URT resulted in differential improvements in specific UE/trunk performance in patients with stroke. BRT elicited larger benefits than URT on reducing compensatory trunk movements at the beginning of reaching. In contrast, URT produced better improvements in UE temporal efficiency. These relative effects on movement kinematics, however, did not translate into differential benefits in daily functions.

**Trial registration:**

ClinicalTrials.gov: NCT00917605.

## Background

Stroke is the most common cause of permanent disability worldwide [1]. Many stroke survivors encounter complex neurologic deficits, leading to poor movement quality, muscle weakness, sensory dysfunction, and cognitive impairments [2]. Approximately 80% of patients with stroke experience upper extremity (UE) paresis [3], causing deficits in motor control [4] and motor function [5] and, consequently, have limitations in daily function [6].

The principles of neurologic rehabilitation are primarily derived from motor learning theories [7]. Several principles have been proposed for better treatment outcomes in stroke patients, including high-intensity, task-specific activities, high repetition [8-10], and active patient participation in treatment activities [11]. Neurorehabilitation principles have been used to develop two different types of arm training in stroke rehabilitation: unilateral and bilateral training. Unilateral arm training emphasizes the mass practice of the paretic arm, such as constraint-induced therapy [12]. Bilateral arm training refers to symmetrically bilateral movement with repetitive practice, such as movement practice with rhythmic auditory cueing training [13].

Both types of arm training have been investigated in empirical research; however, one of the major challenges in implementing the arm training protocols in standard stroke rehabilitation settings is the demand of one-to-one therapist guidance. Labor-intensive UE therapies can be delivered efficiently by robotic devices [11,14,15]. Examples of unilateral robotic devices include the Massachusetts Institute of Technology (MIT)-Manus [16] and the Assisted Rehabilitation and Measurement (ARM) Guide [17], and bilateral devices include the Bi-Manu-Track [18] and the Mirror-Image Motion Enabler (MIME) [19].

An increasing number of efficacy studies [20-22] and review articles [14,15] have shown that robot-assisted arm training improves UE recovery (i.e., motor control and motor function) in stroke survivors. Unfortunately, these review studies did not compare the relative effects of unilateral vs. bilateral robotic training. The bilateral and unilateral approaches to robot-assisted arm training may produce differential effects [23]. Very limited clinical trials have compared the effects of using a robotic device [15] and warrant further scrutiny [24]. In addition, the meta-analysis by Kwakkel and colleagues [15] pointed out that the study [22] using the Bi-Manu-Track for bilateral arm training elicited a larger effect size compared with other studies that mostly used unilateral arm training protocols. Moreover, this study also demonstrated that the bilateral arm trainer resulted in motor recovery not only on the distal part (i.e., wrist and hand) of the UE but also on the proximal part (i.e., shoulder and elbow) in stroke patients. These findings imply that the Bi-Manu-Track is a promising device for further exploring the specific effects of robotic-assisted training on various domains.

The Bi-Manu-Track robotic device was originally designed to provide synchronous bilateral arm training [25] but might also serve as a unilateral training device. One preliminary study [26] modified the bilateral modes of the Bi-Manu-Track to unilateral modes. The results showed that unilateral training might be more effective in enhancing motor impairment, distal muscle power, and grip strength, whereas bilateral training might uniquely improve proximal muscle power. Further research with a larger sample is needed to assess the relative effects of these two training approaches on other domains of treatment outcomes important for stroke motor rehabilitation, including arm and trunk control and daily functions.

Several studies have used functional task protocols to compare the relative effects of unilateral vs. bilateral arm training [27-35], and debate continues whether and in which cases one approach could be superior to the other [23,24]. Three studies [27-29] revealed that unilateral arm training produced greater functional gains and use of the paretic arm in daily life, whereas bilateral arm training improved proximal upper limb motor impairment and force generation or increased movement smoothness. However, other studies [31,32] have suggested that both approaches had comparable effects on motor function in patients with stroke. Accordingly, a comparative study investigating unilateral and bilateral arm training, especially based on a robotic device, is necessary to clarify the possible differential effects between the two types of arm training.

Given that the cerebral motor cortex is responsible for motor control of the UE and that the design of the primary control strategies of the central nervous system are based on hand and movement end-point coordinates [36], the damaged motor cortex after stroke results in some common deficits in the end-point control strategies, characterized by decreased temporal efficiency and smoothness of movement [4]. To overcome these impairments when performing functional tasks, stroke survivors may rely on compensatory strategies of the trunk [37], thus limiting the capacity for subsequent motor gains of the paretic arm [37,38]. Because the trunk acts as a posture stabilizer in reaching activities and is involved in the reaching process, it is important to include an analysis of trunk movement in forward reaching [39]. Kinematic analysis can provide more specific information about control strategies [4] and distinguish between compensation with the trunk and the reappearance of premorbid patterns [37]. Use of kinematic information may provide a better understanding of how specific robot-assisted arm training influences the recovery of movement.

The purpose of our research was to study the relative effects of a unilateral (URT) vs. a bilateral robot-assisted arm training (BRT) protocol using the Bi-Manu-Track on movement control and compensation, motor function, and daily function in patients with chronic stroke. We hypothesized from the results of previous studies using functional task protocols [27-29] that URT might produce greater benefits on motor function and daily use of the paretic limb and that BRT might result in better outcomes on motor control.

## Methods

This study was approved by the Institutional Review Board, and signed informed consent forms were obtained from all participants.

### Participants

We recruited 53 patients (35 men and 18 women) with unilateral stroke identified by brain imaging. Eligibility criteria included first clinical stroke diagnosis, status 6 months to 5 years poststroke, mild-to-moderate motor impairment (score of 20 to 66 on the FMA for the upper limb) [19,40], no severe spasticity in any joints of the paretic arm (Modified Ashworth spasticity score ≤ 3) [41], no serious cognitive deficits (Mini-Mental State Evaluation score ≥ 22) [42], and no participation within the past 3 months in any experimental rehabilitation or drug studies.

### Design overview

**Figure 1:**
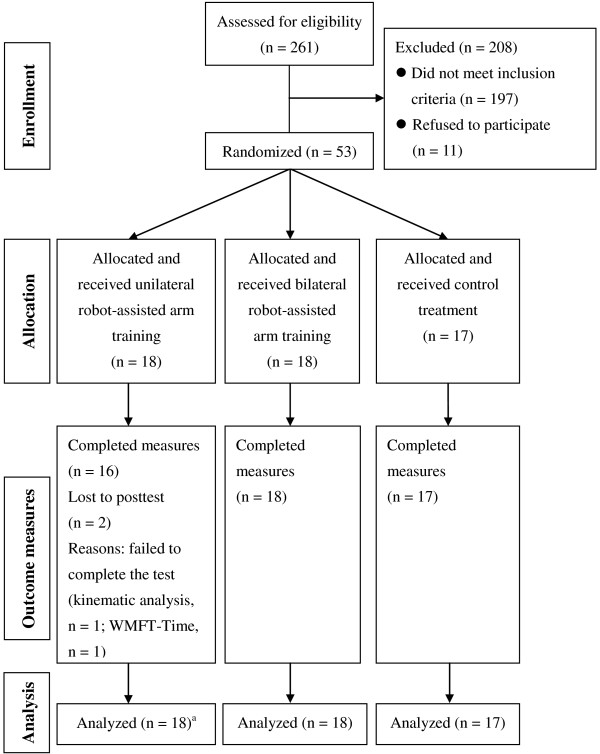
**Flow diagram shows the randomization process.**^a^Missing data were imputed using the group mean. WMFT, wolf motor function test.

This was a randomized pretest and posttest control group design in which the 53 participants were randomized to URT, BRT, or control treatment (CT). Figure 1 shows the CONSORT flow diagram. A prestratification strategy was applied according to the severity of motor impairment (FMA for upper limb total score: 26–40 vs. 40–66) [19] to ensure the participants were equally distributed. Patients were blinded to the study hypotheses.

The 3 groups received the intervention by certified occupational therapists for 90 to 105 minutes per session, 1 session each weekday, for 4 weeks. Clinical evaluations were administered before and after the 4-week intervention by 2 certified and trained occupational therapists blinded to the study group. Kinematic evaluation was conducted by the same research assistant. The study was conducted between January 2010 and March 2011.

### Intervention

The Bi-Manu-Track (Reha-Stim Co, Berlin, Germany) robotic arm trainer was used for treatment. The Bi-Manu-Track enables 2 mirror-like movements: forearm pronation–supination and wrist flexion–extension. Three computer-controlled modes were programmed. In mode 1 (passive–passive mode), the patient’s hands were guided passively by the machine. In mode 2 (active–passive mode), the patient’s nonparetic hand drove the paretic arm in a symmetric direction. In mode 3 (active–active mode), the arm trainer provided resistance to both arms, such at the nonparetic arm had to overcome continuous resistance through the entire movement and the paretic arm overcame only the initial resistance, which was set by the therapist.

The participants sat at a height-adjustable table, with elbows bent at 90°, placed in the midposition into the arm troughs, and with hands grasping 3-cm-diameter handles so that the movement practiced was restricted to the arm and did not involve the trunk. A computer game (e.g., picking up and placing apples to make apple jam) was used along with the robotic device to facilitate patient participation and motivation.

The URT and BRT included 75 to 85 minutes of robot-assisted arm training during which each patient practiced 300 to 400 repetitions of the elbow and wrist cycles in mode 1 and mode 2 and practiced 50 to 80 repetitions in mode 3. This was followed by 15 to 20 minutes of functional tasks practice that included unilateral and bilateral tasks and was added to the 3 training protocols in an attempt to facilitate transfer of reacquired skills to daily activities. Some examples of unilateral training were reaching to grasp a cup, picking up coins, and putting pegs into holes on a board. Examples of bilateral functional tasks training included wiping a table with 2 hands, picking up 2 pegs, opening a box with 1 hand stabilizing while the other hand manipulated, and scooping beans from a bowl with 1 hand while the other hand held the bowl.

#### The URT group

**Figure 2:**
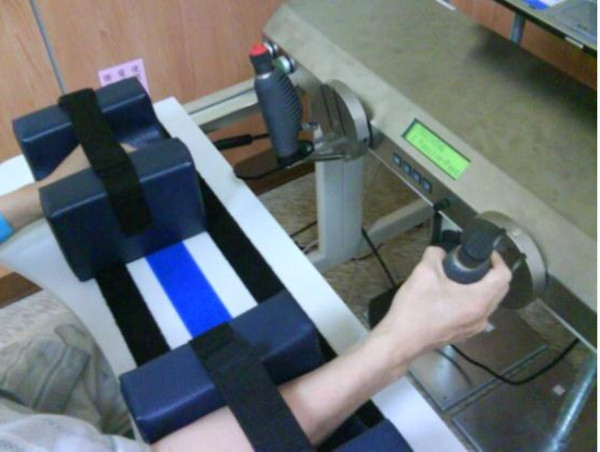
Unilateral robot-assisted training is shown with the Bi-Manu-Track.

Because patients in the URT group practiced only with their paretic arm, we modified the 3 modes: during mode 1 training, the robotic device provided full assistance for the paretic arm; during mode 2 training, the paretic arm moved the handle independently; and during mode 3 training, the paretic arm moved the handle against a resistance determined by the therapist through the entire movement (Figure 2).

#### The BRT group

**Figure 3:**
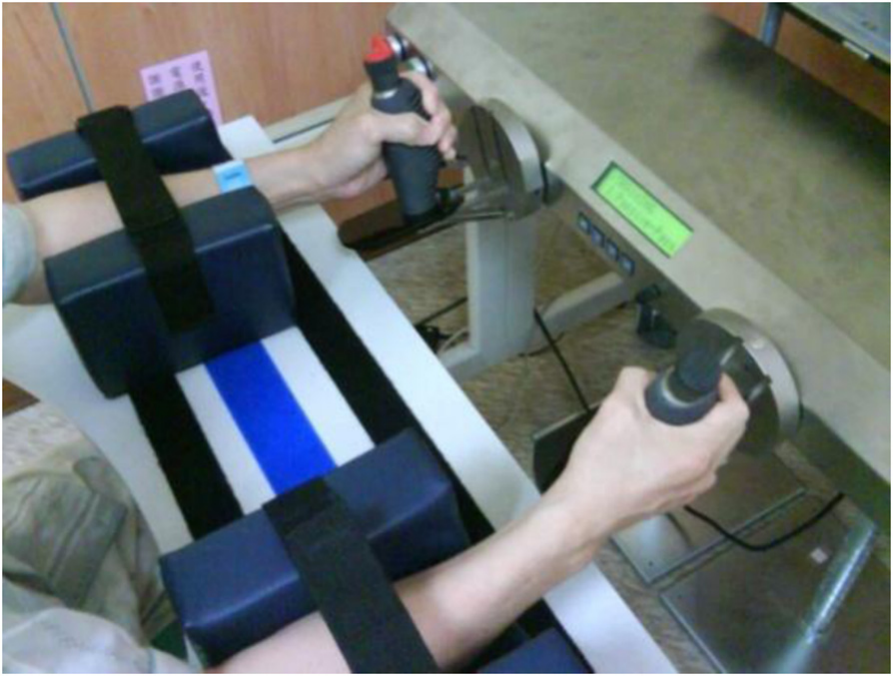
Bilateral robot-assisted training is shown with the Bi-Manu-Track.

Three modes set by the Bi-Manu-Track were used in this group. For mode 3, the therapists determined resistance according to the one that the patient performed the voluntary movement with maximal force against (Figure 3).

#### The CT group

The CT was designed to control for the duration and intensity of the robot-assisted arm training and allowed us to estimate if robot-assisted arm training would confer greater benefits than the CT conventionally used in the clinics. The therapeutic activities in the CT group included weight bearing, stretching, and strengthening of the paretic arm, coordination tasks, unilateral and bilateral fine motor tasks, and balance activities.

### Outcome measures

#### Primary outcome measure—kinematic analysis

Experimental tasks included 1 unilateral and 1 bilateral task. Participants sat in a height-adjustable, straight-back chair with seat height set to 100% of the lower leg length. In the initial position, the tested arm was pronated and the hand was placed on the edge of the table in a neutral position with 0° flexion at the shoulder joint and 90° flexion at the elbow joint.

The targets were a desk bell (3 cm in length and width, and 0.5 cm in height) placed in the midsagittal plane in the unilateral condition and 2 desk bells in front of the bilateral acromia in the bilateral condition. We measured the arm length as the distance from the medial border of the axilla to the third fingertip and standardized the reaching distance to 125% of this distance. For the unilateral task, participants were instructed to use the index finger of the paretic hand to press the bell as fast as possible. For the bilateral task, participants were instructed to use the index fingers of both hands to press the bells simultaneously as fast as possible.

**Figure 4:**
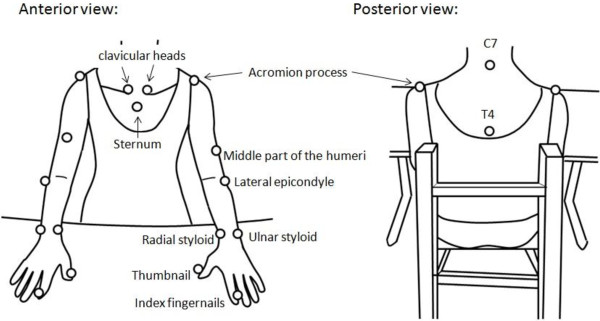
**Schematic diagrams (anterior and posterior view) show the upper limb model used for the 3-dimensional markers that were placed on the skin.** (○): Retroreflective markers placed on the skin.

Three-dimensional motion analysis was performed with a 7-camera VICON MX system (Oxford Metrics Inc, Oxford, UK) recording at 120 Hz and connected to a personal computer to capture the movement of 19 markers. The markers were placed on the sternum, spinal process (C7 and T4), bilateral thumbnails, index fingernails, ulnar styloids, radial styloids, lateral epicondyles, middle part of the humeri, acromion processes, and clavicular heads (Figure 4).

The system was calibrated to have averaged residual errors not exceeding 0.5 mm for each camera before data acquisition. One channel of analog signals was collected to signal the end of the movement when the bell was pressed. Movement onset was defined as a rise of tangential wrist velocity above 5% of its peak value for both testing tasks. Movement offset was the time when the participant pressed the bell.

#### Data reduction for kinematic variables

The captured data were transferred to LabVIEW (National Instruments, Inc, Austin, TX) software for processing. Because the end-point control deficits and abnormal trunk compensation are critical for stroke manifestation and rehabilitation, kinematic variables were chosen to describe the hand end-point control and trunk compensatory strategies during reaching. Movement time (MT) and movement units (MUs) were obtained to reflect the end-point control in reaching. MT refers to the time for execution of the reaching movement, representing temporal efficiency. Smoothness of movement was characterized by the number of MUs. One MU was defined as 1 acceleration and 1 deceleration phase. MT and MU were normalized (NMT, NMU) to correct for variations in reaching distance among participants [43].

Trunk compensation changes were denoted by the arm-trunk contribution slope, assessed as the ratio of the sagittal displacement [37] between the index marker and the sternal marker to the sagittal displacement of the sternal marker. Lower slope values indicate more trunk displacement (i.e., more trunk compensation involved) [37]. Only sagittal displacement was considered because the experimental task used in this study was limited to forward reaching. Sagittal movements are primarily involved, and movements in the vertical direction are trivial. We divided the reaching movement equally into 3 phases (start, middle, and last) according to MT. Because the contribution of the trunk movement usually occurred earlier in the reach in patients with stroke compared with healthy individuals, only the slope values at the start and middle phases were used as the dependent variables in the present study [37]. Intraclass correlation coefficients (ICCs) were calculated to determine the reliability of the kinematic model applied in this study. The results showed good test–retest reliability for the kinematic variables (ICC = 0.74–0.95).

#### Secondary outcome measures—clinical assessments

Poststroke UE motor function was assessed with the Wolf Motor Function Test (WMFT) [44]. Patients were timed and scored for functional ability on a 6-point ordinal scale as they executed 15 activities that included gross and fine-motor tasks. The average time (WMFT-Time) and functional ability scores (WMFT-FAS) of the paretic arm were reported. Activities of daily living were measured with the Motor Activity Log (MAL) [45] and the ABILHAND Questionnaire [46]. The MAL is a reliable and valid 30-item measure of how patients perceive the use of their paretic UE during real-world activities. Patients use a 6-point ordinal scale (0 = no use, 5 = normal use) to rate the amount of use and quality of movement. The ABILHAND is a Rasch-based assessment that measures how a patient perceives his or her bimanual ability in performing daily activities. It uses a 3-point ordinal scale to rate the difficulty in performing 23 daily activities involving bilateral hand function. Online Rasch software was used to convert the ordinal score into an interval measure [47].

### Data analysis

Data were analyzed with SPSS 14.0 software (SPSS Inc, Chicago, IL, USA). A sample size of at least 39 (13 in each arm) was needed for an 80% likelihood in detecting a group difference with a type I error of .05, as determined from a previous bilateral training study showing a large effect size for improving motor control [29].

Groups were examined for baseline differences by using the χ^2^ or Fisher exact tests for categoric data and analysis of variance for continuous data. Between-group differences in kinematic variables and clinical outcomes were analyzed using analysis of covariance. Pretest performance was the covariate, group was the independent variable, and posttest performance was the dependent variable. Intention-to-treat principle was applied, and missing data were imputed with use of group mean score, as described by Herman et al. [48]. Treatment effect sizes (the magnitude of group differences) were evaluated using the effect size η^2^ (η^2^ = SS_b_/SS_total_). The value of η^2^ is independent of sample size and represents the variability in the dependent variable (posttest performance) that can be explained by group differences. A large effect is represented by an η^2^ of at least 0.138, a moderate effect by an η^2^ of 0.059, and a small effect by an η^2^ of 0.01. Significance was set at *P* < .05 for all analyses [49].

## Results

The demographic and clinical characteristics of participants did not differ significantly at baseline among the 3 groups (Table 1). Tables 2 and 3 report the descriptive statistics and inferential statistics for kinematic variables and clinical measures, respectively. In the URT group, 1 participant each was missing kinematic data for the unilateral task and clinical data for the WMFT-Time. Missing data were imputed using the group mean score. All participants tolerated the training protocols, and no adverse events were reported.

**Table 1:** Characteristics of study participants

**Variable** ^**a**^	**URT (n = 18)**	**BRT (n = 18)**	**CT (n = 17)**	***F***	***P***
Sex				1.35	.51
Male	10	13	12		
Female	8	5	5		
Age, years	54.95 (9.90)	52.21 (12.20)	54.22 (9.78)	0.32	.73
Side of brain lesion				1.60	.45
Right	12	9	8		
Left	6	9	9		
Months after stroke	19.00 (15.51)	23.28 (15.37)	23.41 (15.24)	0.47	.63
MMSE score	27.50 (2.53)	28.00 (2.50)	28.38 (2.00)	0.59	.56
FMA score	42.89 (8.62)	43.11 (9.18)	44.06 (11.04)	0.07	.93

^a^ Categoric data are shown as number, and continuous data as mean (standard deviation).

Abbreviations: URT, unilateral robot-assisted training; BRT, bilateral robot-assisted training; CT, control treatment; MMSE, Mini-Mental State Examination; FMA, Fugl-Meyer Assessment.

**Table 2:** Descriptive and inferential statistics for kinematic variables

**Variable**	**Pretreatment, mean (SD)**			**Posttreatment, mean (SD)**			**ANCOVA**		
	**URT (n = 18)**	**BRT (n = 18)**	**CT (n = 17)**	**URT (n = 18)**	**BRT (n = 18)**	**CT (n = 17)**	***F***	***P***	**η**^**2**^
**Unilateral task**									
NMT (sec/mm)	0.004 (0.002)	0.005 (0.003)	0.004 (0.002)	0.004 (0.003)	0.004 (0.002)	0.003 (0.001)	0.57	.57	0.023
NMUs (unit/mm)	0.03 (0.01)	0.03 (0.03)	0.02 (0.01)	0.02 (0.02)	0.02 (0.01)	0.01 (0.01)	0.96	.39	0.038
Trunk contribution									
Slope: start	1.80 (1.05)	2.29 (1.54)	2.65 (1.55)	1.29 (1.09)	2.24 (1.39)	2.50 (1.45)	2.47	.10	0.092
Slope: mid	0.71 (0.41)	0.75 (0.56)	0.81 (0.43)	0.89 (0.51)	0.80 (0.52)	0.93 (0.54)	0.42	.66	0.017
**Bilateral task**									
NMT (sec/mm)	0.008 (0.01)	0.006 (0.004)	0.005 (0.003)	0.005 (0.003)	0.005 (0.002)	0.004 (0.003)	0.33	.72	0.013
NMUs (unit/mm)	0.04 (0.06)	0.04 (0.04)	0.02 (0.02)	0.02 (0.02)	0.03 (0.01)	0.02 (0.02)	0.27	.77	0.011
Trunk contribution									
Slope: start	0.75 (0.53)	1.11 (0.61)	1.06 (0.66)	0.51 (0.90)	1.66 (1.46)	1.60 (1.11)	3.32	.045^a,b^	0.120
Slope: mid	0.52 (0.71)	0.30 (0.45)	0.66 (0.65)	0.90 (0.67)	0.44 (0.59)	0.34 (0.61)	5.78	.01^a,c^	0.190

Abbreviation: SD, standard deviation; ANCOVA, analysis of covariance; URT, unilateral robot-assisted training; BRT, bilateral robot-assisted training; CT, control treatment; NMT, normalized movement time; NMUs, normalized movement units.

^a^Statistically significant (*P* < .05).

Post hoc analyses: ^b^BRT vs. URT, *P* = .027; CT vs. URT, *P* = .032. ^c^URT vs. CT, *P* = .001.

**Table 3:** Descriptive and inferential statistics for clinical measures

**Variable**	**Pretreatment, mean (SD)**			**Posttreatment, mean (SD)**			**ANCOVA**		
	**URT (n = 18)**	**BRT (n = 18)**	**CT (n = 17)**	**URT (n = 18)**	**BRT (n = 18)**	**CT (n = 17)**	***F***	***P***	**η**^**2**^
WMFT									
Time	6.14 (3.83)	7.89 (0.57)	5.99 (3.52)	4.51 (2.21)	8.79 (7.57)	5.26 (3.26)	3.58	.04^a,b^	0.13
FAS	3.14 (0.83)	2.83 (0.57)	3.35 (0.83)	3.30 (0.82)	3.06 (0.63)	3.51 (0.79)	0.50	.61	0.02
MAL									
AOU	0.54 (0.49)	0.76 (0.87)	1.03 (1.24)	0.77 (0.68)	0.95 (0.96)	1.32 (1.40)	0.24	.79	0.01
QOM	0.63 (0.68)	0.79 (0.76)	1.08 (1.09)	0.92 (0.87)	0.99 (0.89)	1.33 (1.12)	0.46	.64	0.018
ABILHAND	−0.18 (1.09)	−0.23 (1.11)	0.06 (1.43)	0.22 (1.18)	−0.15 (0.90)	0.53 (1.25)	2.20	.12	0.082

Abbreviation: SD, standard deviation; ANCOVA, analysis of covariance; URT, unilateral robot-assisted training; BRT, bilateral robot-assisted training; CT, control treatment; WMFT, Wolf Motor Function Test; Time, performance time; FAS, functional ability scores; MAL, Motor Activity Log; AOU, amount of use; QOM, quality of movement; ABILHAND, ABILHAND Questionnaire.

^a^ Statistically significant (*P* < .05).

Post hoc analyses: ^b^URT vs. BRT, *P* = .011.

### Primary outcome measure—kinematic analysis

Arm motor control did not differ significantly in NMT and NMU variables in unilateral and bilateral tasks among the 3 groups. For trunk compensatory strategies, the trunk contribution slope for the start and middle parts represented significant differences in the bilateral task among the 3 groups (start part: *F*_2,49_ = 3.318, *P* = .045, η^2^ = .119; middle part: *F*_2,49_ = 5.783, *P* = .006, η^2^ = .191; Table 2). Post hoc analyses revealed that the BRT and the CT groups demonstrated a larger value of trunk contribution slope for the start part than the URT group (BRT vs. URT, *P* = .027; CT vs. URT, *P* = .032), reflecting that the BRT and CT groups experienced significantly less trunk compensation compared with the URT group. Furthermore, no significant difference was found between URT and BRT for the middle part, and URT produced significantly a greater slope value than CT (*P* = .001), meaning that less trunk compensation was observed in the URT group than in the CT group.

### Secondary outcome measures—clinical measures

The 3 groups did not differ significantly in the WMFT-FAS, MAL, and ABILHAND scores (Table 3); however, significant differences were found in WMFT-Time (*F*_2,49_ = 3.578, *P* = .035, η^2^ = .127). Post hoc analyses indicated that the URT group, but not the CT group, showed less WMFT-Time (*P* = .011) than the BRT group, suggesting that significantly better movement efficiency occurred in the URT group than in the BRT group.

## Discussion

A unique aspect of this study is that the effects of URT vs. BRT were compared using the same robotic arm trainer. The relevant effects of URT vs. BRT presented here advance the clinical application of robot-assisted arm training for subgroups of stroke patients who have different training goals. Another unique aspect is that the study examined the effects of an alternative, unilateral use of the Bi-Manu-Track, which is primarily used in a bilateral manner. No adverse events were reported in the URT group, suggesting that the use of the Bi-Manu-Track in a unilateral manner is feasible. More important, this study demonstrated an innovative and beneficial protocol of unilateral practice using the Bi-Manu-Track, supporting the versatility of the device in stroke rehabilitation.

This study demonstrated that URT and BRT had differential benefits on trunk compensation and motor function, partially consistent with our hypothesis. In the bilateral task after treatment, the BRT and the CT groups exhibited less trunk compensation than the URT group for the start part of reaching. However, only the URT group showed less trunk recruitment for the middle part of reaching during the bilateral task compared with the CT group. The URT group also obtained a higher WMFT-Time score than the BRT group, thus demonstrating larger improvements in the temporal efficiency of motor function. Inconsistent with our hypothesis, no differential effects were found on arm motor control and daily function between URT and BRT.

A study by Levin and colleagues [37] demonstrated that the displacement of the trunk movement throughout reaching usually occurred earlier and was greater in patients with hemiparesis than in healthy individuals. The present study showed that after training, the BRT group demonstrated less trunk involvement at the beginning of reaching than the URT group. Comparable effects were obtained between BRT and URT on trunk compensation at the middle part of reaching, and the descriptive data indicated that BRT and URT both decreased compensatory trunk movement, as represented by increased values of the slope at the middle part of reaching after intervention.

The results at the start and middle part of reaching showed that the beneficial effects on lessening trunk compensation appeared greater in the BRT group, which might be partly related to neurologic reorganization in the motor cortex. When both arms execute bilateral symmetric movements simultaneously in the BRT, the “template” generated by the undamaged hemisphere may provide normal motor plans (i.e., reaching with appropriate trunk recruitment) [13,32,37] to assist in restoring the movement pattern of the hemiplegic side [50]. Repetitive practice furthers the reorganization of motor cortex and movement control. Consequently, BRT might lead to less compensatory trunk movements at the beginning of reaching than URT.

Trunk compensation may also be associated with insufficient trunk and proximal muscle strength [37]. Compared with URT, BRT was suggested to engender greater muscle strength in the proximal part of the arm [26,32], which may decrease the need of trunk involvement to assist in performing reaching tasks and lead to the superiority of BRT vs. URT in reducing compensatory strategies.

The superiority of URT to BRT on the WMFT-Time assessment might be because the amount of active training was greater in the URT than in the BRT group. The affected limbs of the patients in the URT group moved actively in mode 2 and moved against resistance through the entire movement in mode 3, whereas the affected limbs of the patients in the BRT group were guided passively by the unaffected limbs in mode 2 and moved against only initial resistance in mode 3. Studies of motor control suggested that physically assisting a movement may decrease motor learning [51,52] by changing the dynamics of the tasks, reducing the appropriate error signal of motor performance necessary to perform the task successfully, and decreasing motor output and attention during training [53,54]. Shumway-Cook and Woollacott [36] further suggested that active practice or search for optimal strategies is an effective way to learn how to successfully achive a movement goal demanded by the contex. In addition, several functional tasks in the WMFT involve the abilities of wrist and hand of the paretic limb [44]. Given the previous study findings [26,32] that patients with stroke obtained greater gains in muscle strength in the distal part of the paretic arm after URT than after BRT, the URT group may have better motor capacity to efficiently perform the functional activities required in the WMFT.

No between-group differences in the kinematic variables were found in the unilateral tasks. During bilateral simultaneous movements, the interlimb coupling phenomenon (i.e., the movement pattern of one arm influences that of the other) may lead to benefit for the affected arm [55]. Accordingly, bilateral symmetrical tasks may augment the performance of the paretic limb, rendering the improvements in kinematic variables more prominent than in unilateral tasks. Thus, bilateral symmetrical tasks may be more sensitive to detect improvements than unilateral tasks.

The kinematic variables of arm motor control did not differ significantly in NMT and NMUs among the 3 intervention groups. This finding is not consistent with a previous study [27] in which distributed constraint-induced therapy, exclusively emphasizing unilateral training, and bilateral arm training improved movement smoothness represented by NMUs compared with the control group. Movement smoothness might be achieved by sound multijoint coordination [56]. The previous study [27] used multijoint functional tasks for repetitive practice, possibly facilitating movement smoothness, whereas the present study focused on single-joint practice, resulting in no salient effects on movement smoothness. The NMU findings in the present study are also inconsistent with those in the study of McCombe Waller et al. [29], possibly because of different training protocols. They used proximal-part UE training and a mechanical device without computerized control systems, whereas the present study used distal-part UE training with an electronic robot device.

Measures of activities of daily living did not differ significantly between the 2 robot-assisted training groups and the CT group, inconsistent with previous studies [27,28]. The unilateral and bilateral robot-assisted training in the present study focused on repetitions of certain movement cycles. In contrast, the previous studies [27,28] used functional tasks for distributed constraint-induced therapy and bilateral arm training. Robot-assisted arm training with limited degrees of freedom may have limited effects on assisting patients in transferring the obtained motor improvements into daily activities without a transfer package. Although our study combined 15 to 20 minutes of functional tasks practice, the amount of practice might not be sufficient. Further research should emphasize the real-world use of stroke patients of their paretic limbs by incorporating sufficient functional task practice with robot-assisted training to optimize functional improvements.

A few study limitations are of note. First, our study lacked follow-up evaluations. The long-term effects of URT and BRT on movement compensation and motor function await scrutiny based on further study with follow-up. Second, the limited sample size did not allow for subgroup analysis based on the severity of motor function defined by the FMA score. Future research is suggested to recruit a larger sample size to examine the relative effects of URT and BRT in stroke patients with different levels of impairment.

## Conclusion

This is the first study to compare trunk-arm control after URT vs. BRT in stroke rehabilitation. The findings suggest that BRT and URT showed differential improvements in specific UE performance in patients with chronic stroke after 4 weeks of robot-assisted rehabilitation. BRT elicited larger improvements in reducing compensatory trunk movements in targeted reaching. In contrast, URT produced better effects on temporal efficiency in UE movements. BRT might be a compelling approach when the treatment goal targets reducing the compensatory strategy, whereas URT might be an optimal choice if movement efficiency is emphasized. URT and BRT, however, did not effectively improve functional performance. Future research may address the dosing issue of practice on functional tasks in robot-assisted rehabilitation programs to elicit transfer and retention of functional gains. Future research may also study the effects of unilateral combined with bilateral training protocols to optimize the possible benefits of robotic training.

## Abbreviations

BRT: Bilateral robot-assisted training; CT: Control treatment; FMA: Fugl-Meyer assessment; MAL: Motor activity log; MT: Movement time; MU: Movement units; NMT: Normalized movement time; NMU: Normalized movement units; UE: Upper extremity; URT: Unilateral robot-assisted training; WMFT: Wolf motor function test.

## Competing interest

The authors declare that they have no competing interests.

## Authors’ contributions

CYW contributed to conception, experimental design, data interpretation, and helped write the manuscript. CLY contributed to data analysis and interpretation and was involved in drafting the manuscript. MDC was involved in analyzing data and drafting the manuscript. KCL contributed to conception, design, interpretation of the results, and revised the manuscript critically. LLW contributed to data collection and helped to interpret the results. All authors read and approved the final manuscript.
